# Factor structure and validity of the shoulder pain and disability index in a population-based study of people with shoulder symptoms

**DOI:** 10.1186/1471-2474-12-8

**Published:** 2011-01-12

**Authors:** Catherine L Hill, Susan Lester, Anne W Taylor, Michael E Shanahan, Tiffany K Gill

**Affiliations:** 1 Rheumatology Unit, The Queen Elizabeth Hospital, 28 Woodville Rd, Woodville South Australia 5011, Australia; 2 The Health Observatory, University of Adelaide, The Queen Elizabeth Hospital, Woodville, South Australia 5011; 3 Population Research and Outcome Studies Unit, Department of Health, Adelaide, South Australia; 4 Department of Medicine, University of Adelaide, Adelaide, South Australia SA 5000; 5 Rheumatology Unit, Repatriation General Hospital, Daw Park, SA 5041, South Australia

## Abstract

**Background:**

The Shoulder Pain and Disability Index (SPADI) is a self-administered questionnaire that aims to measure pain and disability associated with shoulder disease. The aim of the present study was to investigate the construct validity and factor structure of the SPADI in a population-based study of patients with self-reported chronic shoulder symptoms.

**Methods:**

The North West Adelaide Health Study is a representative longitudinal cohort study of people aged 18 years and over. The original sample was randomly selected and recruited by telephone interview. Overall, 3 206 participants returned to the clinic during the second stage (2004-2006) and were asked to report whether they had pain, aching or stiffness on most days in either of their shoulders. Data was also collected on body mass index and shoulder range of motion (ROM) and demographic factors. The SPADI (numeric rating scale) was administered to participants with shoulder symptoms. Principal components factor analysis, with varimax rotation of factor loadings, was used to assess subscale structure of SPADI. Correlations between the SPADI, shoulder ROM and SF-36 were performed.

**Results:**

Overall, 22.3% of participants indicated that they had pain, aching or stiffness in either of their shoulders. SPADI results were available for 588 of participants with current shoulder symptoms. The internal consistency of the SPADI subscales were high (Cronbach's alpha > 0.92). Two factors, explaining 61.4% of the total variance were extracted by factor analysis. These were interpreted as disability and pain respectively. There was a strong negative correlation between SPADI disability subscale scores and shoulder range of motion. SPADI disability, but not pain, subscale scores were correlated with age.

**Conclusions:**

The SPADI is a valid measure to assess pain and disability in people with shoulder pain in a population-based study. In this setting, the SPADI had a bidimensional structure with both pain and disability subscales.

## Background

The Shoulder Pain and Disability Index (SPADI) is a self-administered questionnaire designed to measure the pain and disability associated with shoulder pathology in the outpatient setting [1]. It consists of 13 items in 2 domains; pain (5 items) and disability (8 items), scored on a visual analog scales, ranging from 0 to 100 (0 = no pain/no difficulty and 10 = worst pain imaginable/so difficult) required help. Each item score is equally weighted, then added for a total percentage score from 0 to 100 (0 = best and 100 = worst). The SPADI was developed by Roach and colleagues in 1991 and initially validated in a sample of 37 male patients with shoulder pathology recruited from an ambulatory care clinic [1]. Since then, the SPADI has been validated in other groups including those with adhesive capsulitis and patients recruited from primary care with shoulder pain and shoulder arthroplasty [2-4]. It has also been demonstrated to be responsive to change in a variety of clinical settings such as shoulder arthroplasty, treatment for adhesive capsulitis and subacromial impingement [3-9] The SPADI is self-administered with completion time documented to be generally between 2 and 5 minutes, with relative ease of scoring [2,10].

Several systematic reviews have been conducted to investigate the psychometric evidence of existing shoulder disability questionnaires [11,12], including the SPADI. These have confirmed the high reliability of the SPADI, its validity in a range of clinical setting and responsiveness to change [12]. These studies have encouraged the use of the SPADI in both clinical and research settings. However, it is known that the clinimetric properties of a questionnaire may vary among different setting and populations [13]. Although one previous study has examined the validity of the SPADI in community volunteers [3], it did not validate the SPADI against a health-related quality of life questionnaire or shoulder range of motion. Nor has there been a study of the SPADI in a population sample using random sampling.

The aim of the present study was to investigate the internal consistency, validity and factor structure of the SPADI in a population-based study of patients with self-reported chronic shoulder symptoms. Specifically, to demonstrate construct validity, we sought to compare the SPADI with (1) SF-36, a generic health-related quality of life questionnaire with established validity and reliability and (2) shoulder range of motion using a valid and reliable approach.

## Methods

### Setting and study population

The North West Adelaide Health Study (NWAHS) was established in 2000 in the North-West region of Adelaide, South Australia. The north-west region of Adelaide comprises approximately half of the population of the city of Adelaide and a third of the population of the state of South Australia. The region also reflects the demographic profile of the state, covering a broad range of socioeconomic areas. The study was designed to assess the prevalence of priority conditions in a population-based community-dwelling cohort. Amongst other conditions, this also included musculoskeletal conditions (arthritis and osteoporosis) and presence of joint pain at different sites (shoulder, hand, low back, hip, knee). Results of the shoulder component of the study have been recently reported [14]. The study was approved by the Human Ethics Committee of the North West Adelaide Health Service. Each participant gave written, informed consent.

Participants for Stage 1 of the study (which was conducted between 2000 and July 2003) were recruited randomly from the Electronic White Pages telephone listings and an initial telephone interview was conducted. Those within each household, who were last to have a birthday and aged 18 years and over, were interviewed and invited to attend a clinic assessment. Overall, 4060 participants were recruited. The methodology is described in detail in Grant et al [15].

Stage 2 of the study was conducted between 2004 and 2006. Participants were contacted and invited to participate in a Computer Assisted Telephone interview (CATI), a self completed questionnaire and a clinic assessment. Overall, 88.1% of the participants completed the telephone questionnaire, 81.2% the self-report questionnaire and 81.0% attended the second clinic assessment.

### Data collection

As part of the CATI assessment, participants were asked if they had ever had pain or aching in their shoulder at rest or when moving, on most days for at least a month and if they had ever had stiffness in their shoulder when getting out of bed in the morning on most days for at least a month. Participants who answered positively to either of these questions were also asked the Shoulder Pain and Disability Index (SPADI) [1,4]. As outlined above, the SPADI consists of 13 questions grouped into two subscales of pain and function, asking about pain and function over the past week. Scores range from 0-100 with higher scores indicating greater impairment. It has been shown to have acceptable test-retest reliability [1]. For this study, the numerically scaled SPADI was used. Although initially used as a self administered clinical index utilizing the visual analogue scale (VAS), a numerically scaled SPADI has been found to be highly correlated to the VAS version and suitable for telephone administration [4]. Participants were also asked to complete the Short Form-36 (SF-36) [16], and a range of demographic information was also collected.

As part of the clinic assessment, height and weight were measured and range of movement of both shoulders was assessed for flexion, abduction and external rotation using a Plurimeter V inclinometer [17]. Shoulder range of motion was measured by clinic staff, who were trained by an anthropometrist. Using a published protocol for shoulder range of motion which included standardised starting positions, instructions and placement of inclinometer [17], During the study, there was periodic checking by external assessor for reliability.

### Statistical analysis

Participants were included in this analysis if they had current shoulder symptoms (i.e. a non-zero SPADI score), and answered all of the SPADI questions. Data were analysed using Statistica v6.1 (StatSoft, Inc, Tulsa, OK, USA). Internal consistency was evaluated, for the total SPADI and each SPADI subscale, using Cronbach's alpha, SPADI validity was evaluated by both factor and construct analyses. Principal components factor analyses, with varimax rotated and normalised factor loadings, was used to evaluate and interpret SPADI factor structure. Cross-sectional construct validity analyses was performed by correlation (Pearson's) analysis between SPADI scores and the physical (PCS) and mental component summary scores (MCS) of the SF-36, and 3 measures of shoulder range of movement (abduction, flexion, external rotation, worst shoulder scores).

## Results

Seven hundred and fourteen (22.3%) of NWAHS participants indicated that they had ever pain, aching or stiffness in either of their shoulders. Of these 15/714 (2.1%) were excluded due to incomplete SPADI data and a further 111/714 (15.5%) were excluded as they had no current shoulder symptoms. Therefore, 588 participants were included in the analysis. The patient and baseline characteristics of these participants are outlined in Tables 1 and 2.

**Table 1:** Patient characteristics (N = 588)

Characteristic	%
**Gender**	
Male	41%
Female	59%
**Age (mean, range)**	56 (22-93) years
**Highest education level obtained**	
Secondary	53%
Trade/Apprentice/Certificate/Diploma	36%
Bachelor degree or higher	9%
Other/Not stated	2%
**Employment Status**	
Full time employment	32%
Part time/casual employment	16%
Unemployed	3%
Home duties	14%
Retired	31%
Other/Not stated	4%
**BMI (kg/m**^**2**^**)**	
BMI < 25	27%
BMI 25-<30	35%
> = 30	38%

**Table 2:** Baseline characteristics of study participants

Patient Characteristics	N	Mean (Range)
SPADI pain subscale	588	34.5 (0, 100)
SPADI disability subscale		21.7 (0, 100)
SPADI Total		26.6 (0.8, 100)
Shoulder range of motion (worst shoulder score)		
abduction	531	123 (10, 177)
flexion		138 (10, 180)
external rotation		44 (0, 90)
SF-36 PCS	538	39.6 (10.9, 65.8)
SF-36 MCS		50.6 (9.2, 70.1

### Internal consistency

The scale showed a high degree of item reliability with high Cronbach's alpha scores for both the pain subscale (5 questions, α = 0.85), disability subscale (8 questions, α = 0.90) and total SPADI score (13 questions, α = 0.92)

### Principal components factor analysis

**Figure 1:**
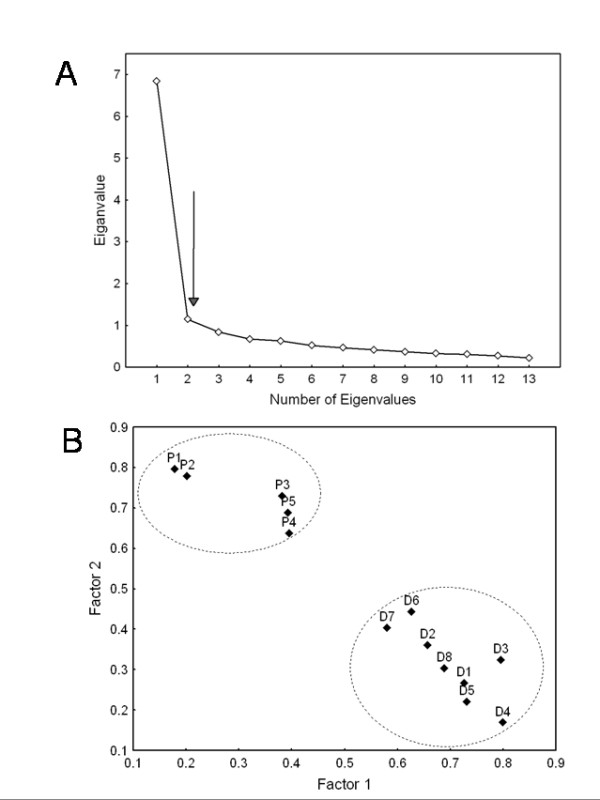
**Principal components factor analysis of the thirteen SPADI items (N = 588)**. (A) Scree plot of eiganvalues: indicating two factors with eiganvalues >1. These two factors collectively explain 61.4% of the total variance. (B) Plot of varimax rotated, normalized factor loadings for each SPADI item: Factor 1, which explains 34.6% of the total variance, is interpreted as disability. Factor 2, which explains 26.8% of the total variance, is interpreted as pain.

The scree plot of the eiganvalue analysis is depicted in Figure 1A. Using the criterion of an eiganvalue > 1, two factors, were subsequently extracted. These two factors explained 34.6% and 26.8% of the total variance respectively, and combined explained 61.4% of the total variance in SPADI scores.

The factor structure was interpreted using varimax rotated, normalised factor loadings (Table 3, Figure 1B). All of the pain items loaded on factor 2 with a coefficient of 0.64 or over. All of the 8 disability items loaded on factor 1 with a coefficient of over 0.58. Therefore, factor 1 is interpreted as disability, and factor 2 is interpreted as pain.

**Table 3:** Varimax rotated, normalised two-factor loadings for the thirteen SPADI items obtained by principal components factor analysis (N = 588)

	Factor 1	Factor 2
Pain section		
PS.1 At its worst?	0.18	**0.80**
PS.2 When lying on the involved side?	0.20	**0.78**
PS.3 Reaching for something on a high shelf?	0.38	**0.73**
PS.4 Touching the back of your neck?	0.39	**0.64**
PS.5 Pushing with the involved arm?	0.39	**0.69**
Disability section:		
DS.1 Washing your hair?	**0.73**	0.27
DS.2 Washing your back?	**0.66**	0.36
DS.3 Putting on an undershirt or pullover sweater?	**0.80**	0.32
DS.4 Putting on a shirt that buttons down the front?	**0.80**	0.17
DS.5 Putting on your pants?	**0.73**	0.22
DS.6 Placing an object on a high shelf?	**0.63**	0.44
DS.7 Carrying a heavy object of 10 pounds?	**0.58**	0.40
DS.8 Removing something from your back pocket?	**0.69**	0.30
Proportion of total variance explained	34.6%	26.8%

### Construct validity

The correlations between shoulder range of motion, PCS and MCS components of the SF-36 and the SPADI are shown in Table 4. These demonstrate moderately strong negative correlations between SPADI disability scores and shoulder range of motion, particularly abduction (r = -0.56) and flexion (r = -0.56). Weaker correlations were observed between shoulder range of motion and SPADI pain scores. There was also a substantial negative correlation between SF-36 PCS scores and SPADI disability scores (r = -0.48, Table 4), whereas SPADI correlations with MCS scores were generally weaker. Further, the SPADI disability score was correlated with age (r = 0.23, p < 0.0001), whereas the SPADI pain score was not (r = 0.02, p = 0.72).

**Table 4:** Correlations (Pearson's r) between SPADI subscales and shoulder range of motion (worst shoulder scores, N = 531), and SF-36 physical (PCS) and mental (MCS) component subscales (N = 538).

	SPADI PAIN	SPADI DISABILITY	SPADI TOTAL
Shoulder flexion	-0.35	-0.56	-0.51
Shoulder abduction	-0.37	-0.56	-0.52
Shoulder external rotation	-0.26	-0.32	-0.32
PCS of SF-36	-0.34	-0.48	-0.46
MCS of SF-36	-0.17	-0.26	-0.24

All p-values < 0.0001

## Discussion

This study provides evidence for the use of the SPADI as a measure of shoulder pain and disability in population studies. Although previous studies have examined the validity of the SPADI in community patients, these have generally been recruited via newspaper advertisements, clinic posters or from primary care [3,4,10], rather than random community sampling as was used for recruitment in the NWAHS cohort study. This study represents the largest study examining the validity of the SPADI and the first in a sample of people with shoulder pain randomly selected from the community. As expected, the median SPADI scores in this population study were lower than in other studies with patients recruited from clinical settings, including both primary or secondary care. It demonstrates the utility of the SPADI within this population setting as it had a high internal consistency, excellent response rate (97.9%) and good construct validity. Further work is needed to determine whether the SPADI is responsive to change within this study setting.

In the initial validation of the SPADI, Roach et al conceptualised 2 subscales measuring shoulder 'pain' and 'disability' [1]. Although the principal component analysis with varimax rotation loaded on 2 factors, these did not delineate clearly between the pain and disability items, with a mixture of pain and disability subscale questions distributing into each factor. However, that study was underpowered as it was performed in only a small group of 37 male veterans. Other validity studies have found the SPADI to be unidimensional in patients with adhesive capsulitis [18] and patients recruited from orthopaedic clinic [19]. Although in the latter study, only unrotated factor analysis was performed. A further analysis of the Turkish translation of the SPADI extracted 3 dimensions [20]. However, several other studies in community practice have confirmed suggested it has 2 dimensions [3,21], albeit with 2 factors that seemed to distribute equally to both subscales. The factor analysis we undertook in this population demonstrates that the SPADI does have a bidimensional structure in this study setting, with the clear interpretation of separate 'pain' and 'disability' subscales. This is in keeping with the initial conceptualization by Roach et al [1]. Both 'pain' and 'disability' subscales have construct validity in that they differ in the correlation with other relevant measures such as SF-36 and shoulder range of motion, supporting the concept of separate pain and disability subscales.

This study, like the original validation study and the study by Williams et al confirmed correlation with shoulder range of motion with closer correlation for the disability subscale [1,4]. The correlation coefficients were similar to those seen in the original validation paper [1]. However, a study of the SPADI in 180 primary care patients showed poor correlation between the SPADI and shoulder range of motion (r values 0.09-0.251)[10].

In this study, although we measured shoulder range of motion, we were not able to clinically assess the participants with shoulder symptoms, due to the large number of participants in the cohort study. Therefore, we do not have clinical diagnoses for these participants. However, previous SPADI validation studies in primary care have recruited participants with a variety of clinical shoulder diagnoses [2,4,10], and still demonstrated adequate psychometric properties.

## Conclusions

In conclusion, this large study demonstrates that the SPADI has a bidimensional factor structure representing pain and disability, with adequate internal consistency and construct validity for use in population studies of shoulder symptoms.

## Competing interests

The authors declare that they have no competing interests.

## Authors' contributions

CLH, TKG, AWT and EMS designed the study, TKG and AWT oversaw data collection. SEL and TKG performed the statistical analysis. CLH drafted the manuscript with help from the other authors. All authors read and approved the final manuscript.

## Pre-publication history

The pre-publication history for this paper can be accessed here:

http://www.biomedcentral.com/1471-2474/12/8/prepub
